# Degassing Rhythms and Fluctuations of Geogenic Gases in A Red Wood-Ant Nest and in Soil in The Neuwied Basin (East Eifel Volcanic Field, Germany)

**DOI:** 10.3390/insects9040135

**Published:** 2018-10-05

**Authors:** Gabriele M. Berberich, Martin B. Berberich, Aaron M. Ellison, Christian Wöhler

**Affiliations:** 1 Image Analysis Group, Faculty of Electrical Engineering and Information Technology, Technical University of Dortmund, 44221 Dortmund, Germany; christian.woehler@tu-dortmund.de; 2 IT-Consulting Berberich, 50374 Erftstadt, Germany; mb@berberichweb.com; 3 Harvard Forest, Harvard University, 324 North Main Street, Petersham, MA 01366, USA; aellison@fas.harvard.edu

**Keywords:** *Formica polyctena*, red wood ant, geogenic gases, East Eifel Volcanic field (EEVF), earthquakes, Earth tides

## Abstract

Geochemical tracers of crustal fluids (CO_2_, He, Rn) provide a useful tool for the identification of buried fault structures. We acquired geochemical data during 7-months of continual sampling to identify causal processes underlying correlations between ambient air and degassing patterns of three gases (CO_2_, He, Rn) in a nest of red wood ants (*Formica polyctena*; “RWA”) and the soil at Goloring in the Neuwied Basin, a part of the East Eifel Volcanic Field (EEVF). We explored whether temporal relations and degassing rhythms in soil and nest gas concentrations could be indicators of hidden faults through which the gases migrate to the surface from depth. In nest gas, the coupled system of CO_2_-He and He concentrations exceeding atmospheric standards 2-3 fold suggested that RWA nests may be biological indicators of hidden degassing faults and fractures at small scales. Equivalently periodic degassing infradian rhythms in the RWA nest, soil, and three nearby minerals springs suggested NW-SE and NE-SW tectonic linkages. Because volcanic activity in the EEVF is dormant, more detailed information on the EEVF’s tectonic, magmatic, and degassing systems and its active tectonic fault zones are needed. Such data could provide additional insights into earthquake processes that are related to magmatic processes at the lower crust.

## 1. Introduction 

The seismically active East Eifel Volcanic Field (EEVF) and its adjoining Neuwied basin have been the focus of many vulcanological, geochemical, petrochemical, and tectonic investigations. These have focused on dormant but not extinct volcanic activity [1,2,3]; the present-day NW–SE-directed compressional stress field and its related seismic activity [4,5]; gas composition and chemical tracers of mineral waters [6,7,8]; and mofettes along the Laacher See or at Obermendig [9]. Data collection and monitoring only has been annual, for example, [8] or short-term (4 days; [9]).

Geochemical tracers of crustal fluid for example, carbon dioxide (CO_2_), helium (He), and radon (Rn) can identify buried fault structures in bedrocks [10,11]. Changes in soil gas concentrations reflect heterogeneities linked to soils or tectonic structures, for example, [9,12]. Faults and fracture networks from macro- to micro-scale are preferential pathways of lateral and vertical degassing, for example, [10,13,14,15]. Important mechanisms driving fluid flow and keeping fractures open are compressive stress, volume changes of pore fluid or the rock matrix, and fluid movement or buoyancy [16].

CO_2_ is mainly produced biologically or in equilibrium with carbonate minerals, but also can originate from mantle degassing or metamorphic processes [17]. He is approximately homogeneous in the atmosphere [18] because solid Earth degassing and escape of He from the atmosphere are in equilibrium [19,20]. However, tectonically active zones often exhibit high fluxes of He; active fractures are highly permeable for He when seismic activity is high [10,21]. Rn forms gas mixtures with other gases, such as the Rn–CO_2_ couple, which is considered to be the most probable carrier-gas mechanism for soil Rn [10,22,23]. The distribution of environmental radon is geologically dependent, varying with local conditions across relatively small distances.

We previously described close spatial relationships between red wood-ant nests (*Formica rufa*-group; henceforth RWA) and tectonic fault zones [24,25,26,27,28,29]. He and Rn in RWA nests exceeded atmospheric and background concentrations [24,25], and nests are associated with fault-related CH_4_ degassing [28]. RWA nests also are “hot spots” for CO_2_ emissions in European forests, increasing their heterogeneity of soil C emissions [30,31,32,33]. Wu et al. [34] showed that nests of the ants *Lasius flavus*, *L. niger* and *F. candida* contributed measurable emissions of CO_2_ (7%) and N_2_O (3.4%) from wetland soils.

Here, we describe associations between carrier-trace-gas couples (CO_2_-He and CO_2_-Rn), fluctuations and degassing rhythms, earth tides, and meteorological and tectonic processes, in three different environments: ambient air (AA), soil gas (SG) and RWA nest gas (NG), to help identify unknown degassing faults at the Goloring site in the Neuwied Basin. Bi-weekly sampling was executed during a 7-month campaign (‘7-M’; 1 March–30 September 2016). During one month of this campaign, we intensively sampled SG1-SG7, NG, and AA every 8 h (‘4-W’; 12 July–11 August 2016).

**Figure 1:**
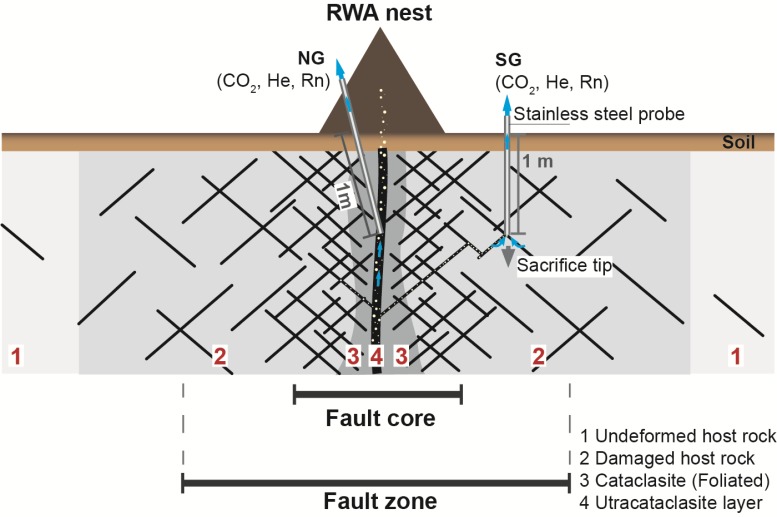
Schematic diagram (not to scale) showing the basic structural elements of a principal fault zone with a red wood ant (RWA) nest (brown triangle) and exemplar nest gas (NG) and soil gas (SG) probes (not to scale). Yellow bubbles indicate gases migrating upward through the fault network. Variation in structure along and between faults is common; damage intensity and thickness of the damaged zone vary laterally towards the core zone. Depending on positions of SG probes within the damaged zone, fluid flow and degassing may be limited (modified after [36]).

We used these associations to test whether: (a) associations between NG and SG concentrations indicated actively in situ degassing faults trapping migrating geogases from deep underground (Figure 1); (b) NG and SG fluctuations in soil and nest were affected by external agents (earth tides, earthquakes, or meteorology); and (c) SG and NG concentrations were associated with those of three nearby degassing mineral springs [35].

We found that NG appears to be associated with SG indicating fault-related micro-seepage of geogases; the degassing rhythm between the soil and nest is associated with degassing rhythms of three nearby mineral springs; and that degassing patterns are independent of earth tides and meteorological conditions.

This study is part III of the research project “GeoBio-Interactions” in which we also monitored geochemistry of three mineral waters ≈6 km from the Goloring site [35] and the association of RWA nests and fault-related CH_4_ degassing at Goloring [28].

## 2. Methods

### 2.1. Study Area

**Figure 2:**
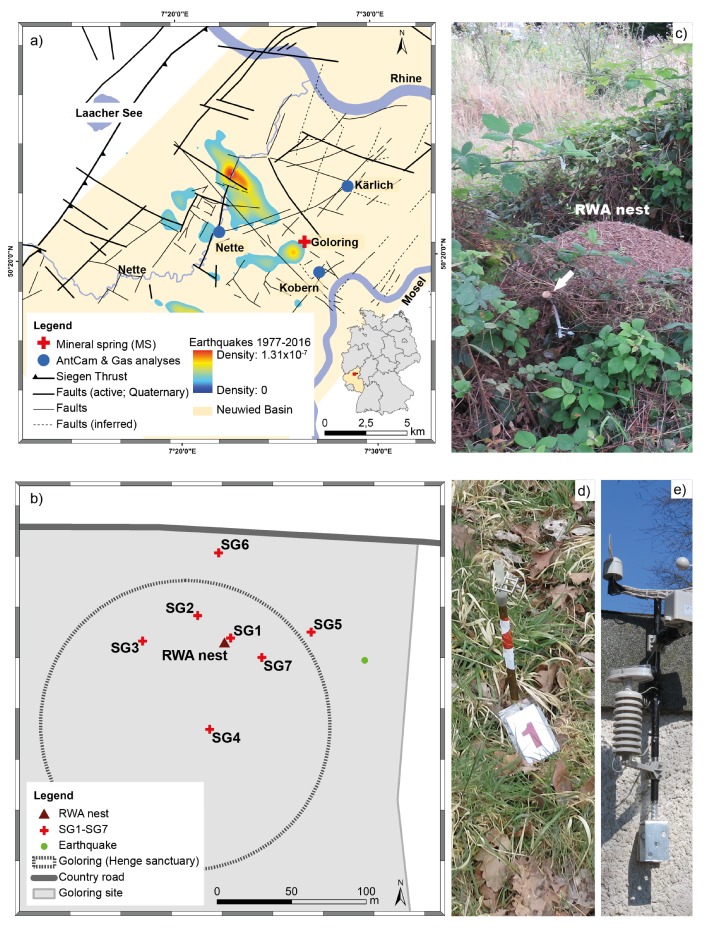
Location of the Goloring study site (red cross) ≈15 km SE of the Laacher See volcano within the Neuwied Basin (light yellow area). The map (**a**) shows tectonic structures (black lines) and probability density of the earthquake events from 1977–2016 related to the Ochtendunger Fault Zone (OFZ; rainbow contours showing the hot spots (red color) of earthquake events within the OFZ rarely exceeding local magnitude of 4.0; modified after [28]). The inset shows the location of the study site within Germany. CO_2_, He, and Rn were sampled at the Goloring site with its Iron-aged henge sanctuary from a RWA nest, soil, and the ambient air; no fault zones had been reported and identified previously from the Goloring study site (**b**). Photographs show (**c**) the permanent nest gas probe (white arrow) and the RWA nest, (**d**) example of a permanent soil gas (SG1) probe with marked flag, and (**e**) the meteorological station (all photographs: G. M. Berberich).

The Goloring site (Figure 2a), with its Iron age henge sanctuary (Figure 2b), is located southeast of the Laacher See volcano, and close to the Ochtendung Fault Zone in the seismically active Neuwied Basin, which is part of the Quaternary East Eifel Volcanic field (EEVF) in western Germany (Figure 2a). During the last 700 ka, intensive intra-continental Quaternary volcanism took place in the EEVF, with its youngest event being a phreato-plinian eruption of the Laacher See volcano ≈12,900 years ago. Today, the volcanic activity is dormant but not extinct [1,3]. Complex major tectonic and magmatic processes, such as plume-related thermal expansion of the mantle-lithosphere [2] and reactivation of Variscan thrust faults due to the present-day compressional stress field oriented in NW-SE direction affect the study area [5]. Weak to moderate earthquakes, which occur mostly in a shallow crustal depth (≤ 15 km) with local magnitudes (M_L_, Richter scale) rarely exceeding 4.0, are concentrated in the seismically active Ochtendunger Fault Zone (OFZ; Figure 2a; [4]). Berberich et al. [28] provide a more complete geological, tectonic, and volcanological description of the Goloring study site. Our Goloring study site is situated in the center of a triangle-shaped study area formed by the three previously-investigated mineral springs (Flöcksmühle in the Nette river near Ochtendung [hereafter: ‘Nette’], Waldmühle in Mülheim-Kärlich [hereafter: ‘Kärlich’] and ‘Kobern’ in Kobern-Gondorf (Figure 2a; [35]). No fault zones had been reported and identified previously from the Goloring study site; local earthquakes magnitude never exceeded M_L_= 2, and focal depths of earthquakes near it never exceeded 28 km during our sampling campaign [37].

### 2.2. Gas Sampling and Geochemical Analyses

We measured gases in ambient air (AA), soil (SG; Figure 2d), and the RWA nest (NG) at the Iron age henge sanctuary at the Goloring site (Figure 2b) biweekly from 1 March–30 September 2016 (7-M; 16 times) and every eight hours between 12 July and 11 August 2016 (4-W; 83 times), yielding a total of 2673 gas samples. Gas sampling followed procedures described by Berberich [24] using analytical equipment and sampling methods described by Berberich et al. [28,35]. Briefly, AA was sampled at 1 m height, 1 m away from the RWA nest. The stainless-steel RWA nest-gas probe (Figure 2b; inner diameter 0.6 cm), equipped with a flexible tip attached to a pushable rod and a sealable outlet for docking sampling equipment, was inserted 1 m into the RWA nest. It remained there, unmoved, during the entire 7-M (including 4-W) sampling campaign. Before the start of the sampling in March, the probe was evacuated twice by pushing the rod using a 20-mL syringe. After this, the outlet was closed to prevent atmospheric influence. Thereafter, the outlet was opened only after docking the sampling unit to it. Seven permanent soil gas probes (Figure 2c), in locations chosen on information from previous investigations, were installed to 1 m depth, either ≈ 2 m (SG1), 30 m (SG 2 and 7), or 60 m (SG 3–6) from the RWA nest (Figure 1c; Hinkle 1994). Occurrences of maximum helium anomalies (> 11 ppm) in SG3, SG4, and SG6 ≈ 60 m away from the nest could be attributed clearly to operator error during analyses.

### 2.3. External Factors

We used data on earthquakes, Earth tides, and meteorological conditions (Figure 2e) (published by Berberich et al. [28].

### 2.4. Data Analysis

All analyses were done using R version 3.3.2 (R Core Team 2016, www.R-project.org) or MATLAB R2017a (www.mathworks.com).

We used the “median + 2MAD” method [38] to separate true peaks in gas concentrations from background or naturally-elevated concentrations: any observation greater than the overall median + 2MAD was considered a peak concentration [38]. For interpreting the significance of the correlation coefficient, we followed Hinkle et al. [39].

Analysis of fluctuations followed Berberich et al. [35]; cross-correlation analyses were used to investigate temporal relations between degassing patterns of sampled springs and carrier-tracer gas relations.

Because meteorological variables were strongly correlated, we used principal component analysis (R function prcomp) on centered and scaled data to create composite “weather” variables (i.e., principal axes) that were used in subsequent analyses.

We used modified Fourier analysis (sampling rate = 8; Matlab 2017a) of the 4-W gas (NG, SG) and Earth-tide data [40] to test for temporal rhythms. Because the observation interval corresponds to an infinite signal multiplied by a rectangular window, a Blackman window [41] was applied to suppress the side lobes of the rectangular window. Because average degassing produced a large peak in the origin of the amplitude spectrum, this peak was removed to reveal any low-frequency components due to the main lobe of the window function [28].

### 2.5. Availability of Data

Data are available from the Harvard Forest Data Archive (http://harvardforest.fas.harvard.edu/data-archive), dataset HF-311.

## 3. Results

### 3.1. Gases in Ambient Air, Soil, and the RWA Nest

#### 3.1.1. CO_2_

Almost all CO_2_ concentrations in NG and SG were above the threshold value (Median + 2MAD) during the 7-M and 4-W campaign. Median values of CO_2_ were different for all environments. The highest median concentrations were found in the soils: SG1 (7-M: 5.30; 4-W: 8.8 Vol. %) and SG5 (7-M: 10.0; 4-W: 12.5 Vol. %); maximum concentrations ranged from 0–15 Vol. %. Median CO_2_ in NG was 0.0–0.4 Vol. %, and it was at 0.0 Vol. % in AA throughout the sampling (Table 1). Three different anomaly classes (after Sauer et al. [42]; Table 2) were identified for CO_2_ concentrations in the environments: (1) < 1 Vol. % (AA and NG), (2) 1–9.99 Vol. % (SG1–4,6,7) and (3) > 10 Vol. %: (SG1, 5).

#### 3.1.2. He

He concentrations higher than the atmospheric standard (5.22 ppm; Davidson and Emerson 1990) indicated tectonic influence were recorded in NG (49%), AA (60%), SG2 and SG3 (≈40%), SG4 and SG6 (≈20%), and SG1, SG5 and SG7 (≈14%; Table 2). In the 7-M samples, maximum He concentrations of ≈5.50 ppm occurred in SG2, 3, and 7 (Table 2). In the 4-W samples, nearly all He concentrations in NG and SG exceeded atmospheric standard; maximum concentrations occurred in SG3, 4, and 6 (>11 ppm), NG (5.85 ppm), and AA (5.54 ppm).

#### 3.1.3. Rn

In the 7-M samples, maximum Rn concentrations in AA, NG, SG3, SG6, SG7 were above the threshold value (Vol. %; Table 1). Peak concentrations occurred in SG4 (163 BqL^−1^), SG1 (138 BqL^−1^), SG5 (100 BqL^−1^) and SG7 (94 BqL^−1^; Table 1). In the 4-W samples, Rn concentrations were highest in SG4 (146 BqL^−1^), SG1 (97 BqL^−1^), SG5 (75 BqL^−1^) and SG 7 (70 BqL^−1^). Rn concentrations in NG (7-M: ≈7 BqL^−1^; 4-W: ≈16 BqL^−1^), SG 2, and SG3 were at or below background levels (Table 2).

### 3.2. Time Series

#### 3.2.1. Fluctuations in Gas Concentrations

**Figure 3:**
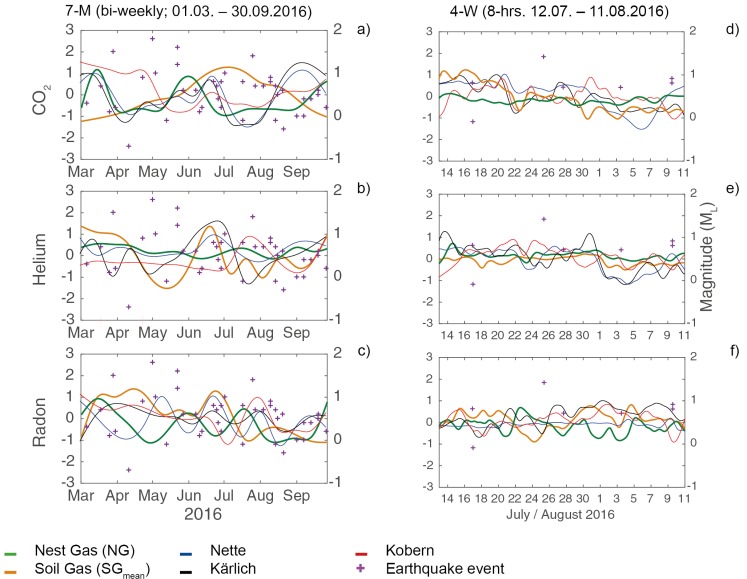
Fluctuation patterns of gas concentrations (centered and scaled data) in (**a**) 7-M and (**b**) 4-W samples in nest gas (NG; green line), soil gas (SG 1-7: SG_mean_; orange line), and mineral springs (other colors; from [35]); earthquake events (M_L_) are indicated with purple crosses.

Gas fluctuation patterns were observed in all samples in all environments (Figure 3). SG and NG concentrations varied by an order of magnitude in the 7-M samples (e.g., CO_2_ in SG5: 6–15 Vol. %; Rn in SG4: 54–163 BqL^−1^; and Rn in NG: 0.1–6 BqL^−1^) and in the 4-W samples (e.g., CO_2_ in NG: 0–11 Vol. %; Rn in SG7: 0.6–69 BqL^−1^), but amplitudes of gas fluctuations were lower in the 4-W samples (Figure 3).

Variations in CO_2_ and He in SG were strongly correlated (*r* = 0.71–0.94; Figure 3a,b,d,e; Appendix B, Figure A3 and Figure A4). High (*r* = 0.81–0.84) to moderate (*r* ≈ 0.63) correlations in Rn variations were observed in SG 1, 2, 5 and 7 (Figure 3c,f; Appendix B, Figure A5). NG and SG fluctuations were moderately correlated (*r* ≈ 0.58) for CO_2_ in the 4-W samples (Appendix B, Figure A3). Moderate correlations (*r* ≈ 0.55) were identified between He variations in AA in the 4-W samples (Appendix B, Figure A4).

#### 3.2.2. Temporal Variations of Concentrations and Carrier-Trace Gas Couples in SG and NG

**Figure 4:**
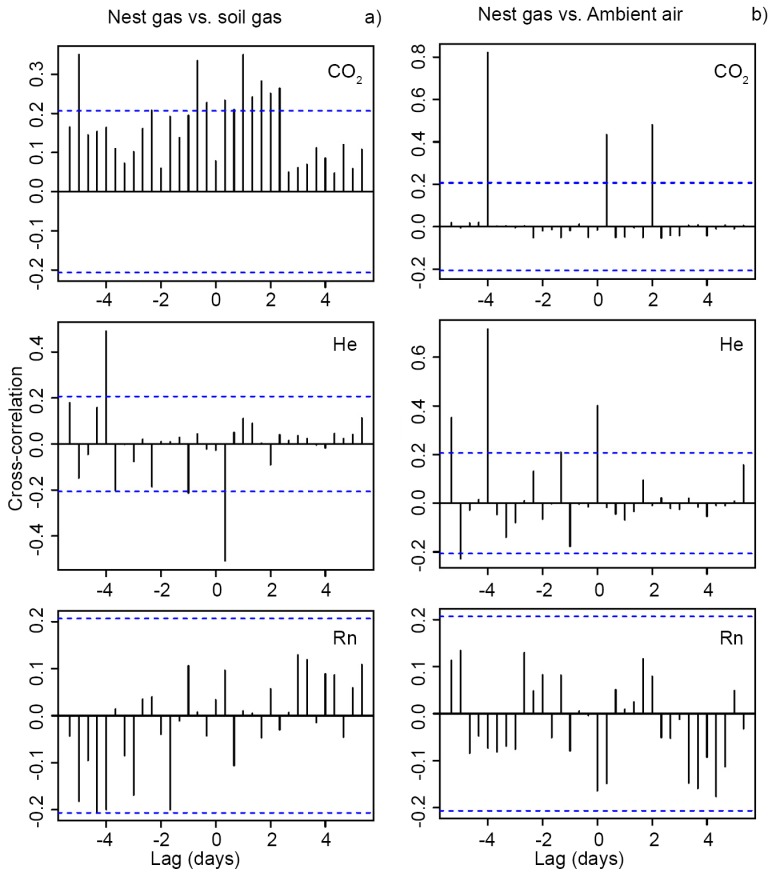
Cross-correlations of the time series of (**a**) NG vs. SG_mean_ and (**b**) NG vs. AA for the 4-W samples. Blue dashed lines indicate confidence thresholds.

Cross-correlations were weakly positive between CO_2_ in NG and SG_mean_ (SG1–7) with a time lag of ≈1 day, and moderately negative between He in NG and SG_mean_ with a time lag of ≈8 h (Figure 4a). Cross-correlations between CO_2_ and He in NG and AA were strong with a time lag of ≈4 days (Figure 4b). No cross-correlations were found for Rn among the samples.

**Figure 5:**
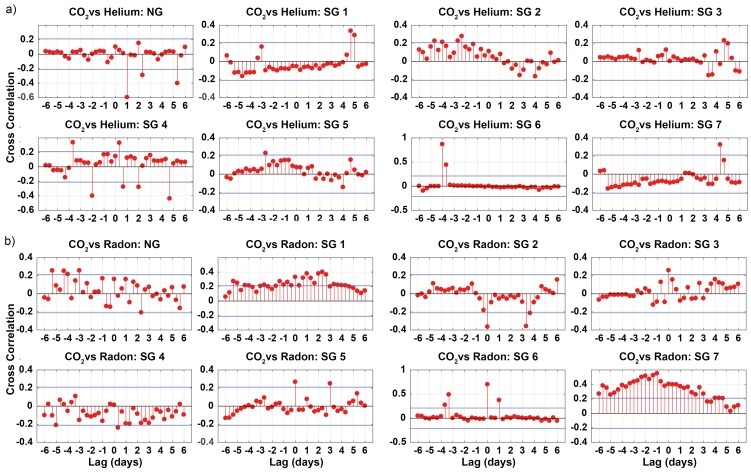
Cross-correlations of time-series of (**a**) CO_2_ vs. He and (**b**) CO_2_ vs. Rn (8-hour median smoothed) in NG and SG from the 4-W samples. Blue dashed lines indicate confidence thresholds.

Joint visualization of the time series of carrier-trace gas couples revealed differences between NG and SG. A coupled system of CO_2_-He is visible in NG (lag = 1 day) and in SG6 (lag = −4 days). The cross-correlation for the CO_2_-He couple in all other SG never exceeded 0.4 (Figure 5a). A CO_2_-Rn coupled system was visible in SG6 (instantaneous) and SG 7 (lag = −1 day). In all other environments, the cross-correlation was low (0.4) for the CO_2_-Rn couple (Figure 5b).

#### 3.2.3. Fourier Analysis

**Figure 6:**
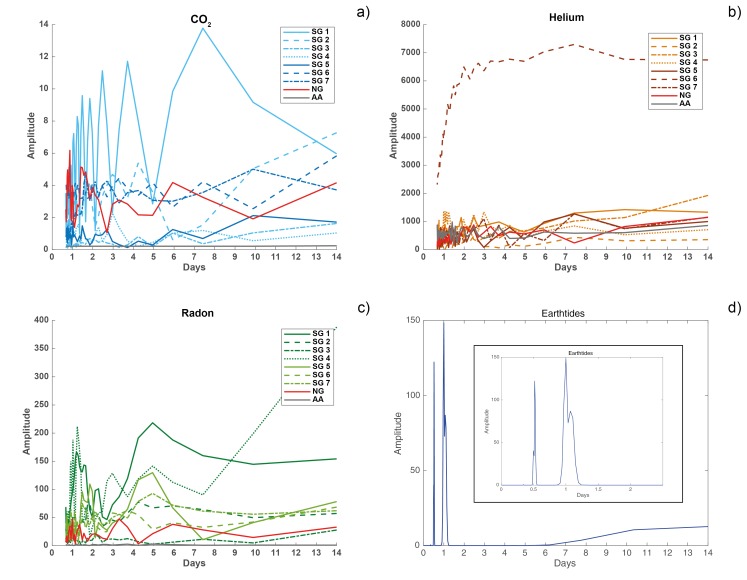
Results of the modified FFT (Fast Fourier Transform) analysis for the degassing patterns of (**a**) CO_2_, (**b**) He, (**c**) Rn in AA, NG and SG 1–SG7 in the 4-W samples and (**d**) earth tides.

Common synchronous infradian degassing rhythms of CO_2_, He, and Rn were observed in the 4-W samples of AA, NG, and SG after 2, 3, 4 and 6 days (Figure 6; Appendix C). There was no common CO_2_ maximum peak (Figure 6a; Appendix C, Table A1). Common He maxima occurred at 7 (NG, SG2), 22 (SG5–SG7), 30 (SG1) and 45 days (SG3; Figure 6b; Appendix C, Table A2), whereas common Rn maxima were observed for 15 days (AA, SG7; Figure 6c; Appendix C, Table A3). At a period of 45 days, Rn maxima were observed only in SG4 (Appendix C, Table A3). Earth tides peaked at periods of 0.5 days and 1 day (Figure 6d).

### 3.3. External Factors

**Figure A1:**
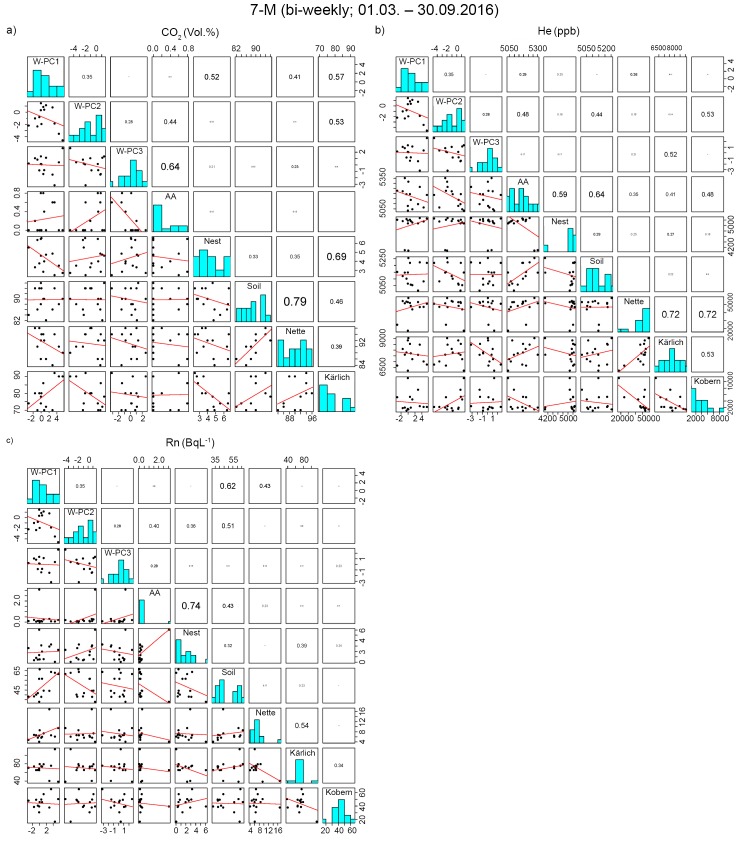
Correlation coefficients of the principal components analysis of weather variables with geogenic gas concentrations for (**a**) CO_2_, (**b**) He, and (**c**) Rn for AA, NG, SG, and the three mineral springs for the 7-M samples; W-PC1 = Weather-PC1.

**Figure A2:**
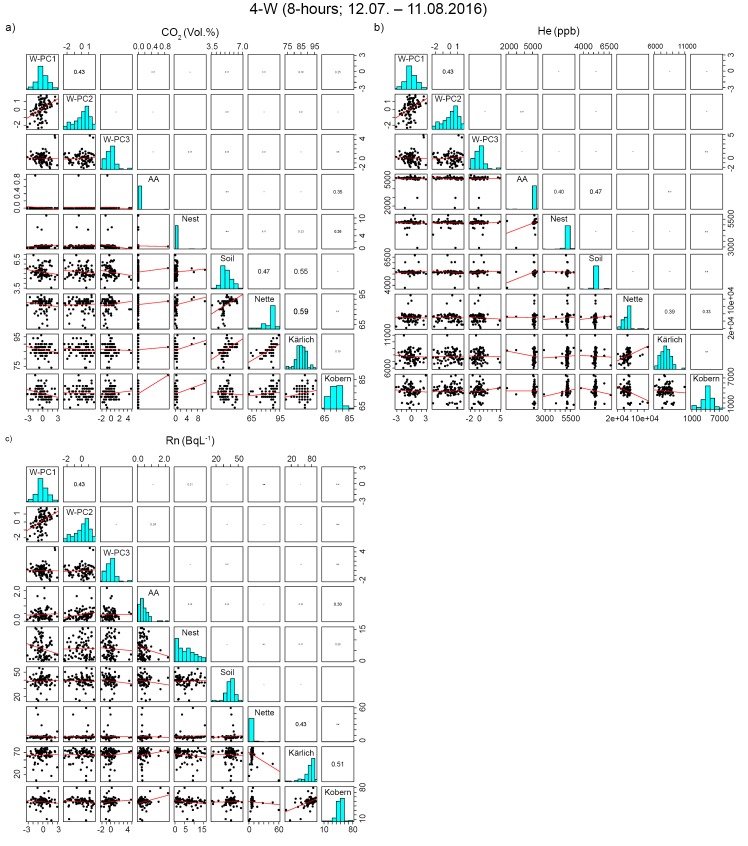
Correlation coefficients of the principal components analysis of weather variables with geogenic gas concentrations for (**a**) CO_2_, (**b**) He, and (**c**) Rn for AA, NG, SG, and the three mineral springs for the 4-W samples; W-PC1 = Weather-PC1.

Stable meteorological conditions persisted during the campaign [28]. The degassing processes from NG and SG did not appear to be associated with meteorological conditions (Appendix A, Figure A1 and Figure A2).

Forty-three small-scale earthquakes (ML −0.7–1.8; depth: 1–26.7 km) occurred during the 7-M sampling interval and five during the 4-W sampling interval (ML −0.1–1.4; depth: 3–11.5 km; [28]). For the 7-M samples, we observed no association between earthquakes and gas concentrations. For the 4-W samples, declines in CO_2_ (SG_mean_), He (NG, SG_mean_), Rn (SG_mean_) fluctuation were observed visually before the earthquake at Nickenich on 3 August (ML 0.7; Depth 7 km) 11.7 km from the Goloring site (Figure 3d–f). After this earthquake, a significant rise in Rn fluctuation was observed in SG_mean_ and NG (Figure 3f).

**Figure A3:**
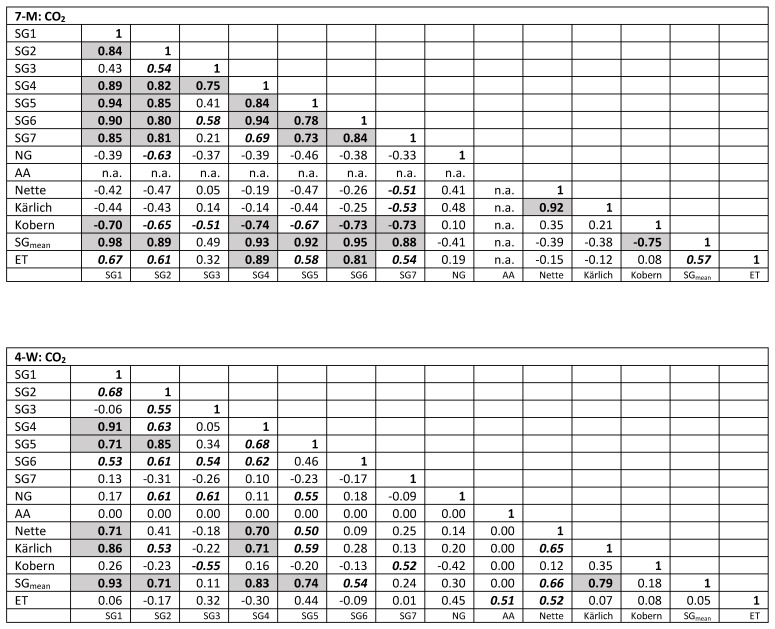
Correlation coefficients between CO_2_ fluctuation patterns in NG, SG, SG_mean_, mineral springs, and Earth tides (ET) for the 7-M and 4-W samples. Correlations >0.70 are shaded. Correlations 0.5–0.7 are marked in bold-italic; n.a.: not available.

**Figure A4:**
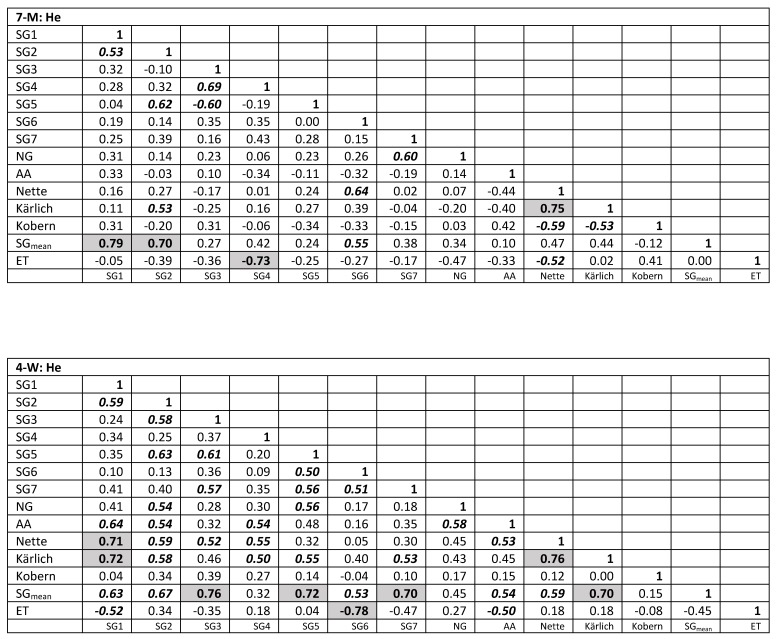
Correlation coefficients between He fluctuation patterns in NG, SG, SG_mean_, mineral springs, and Earth tides (ET) for the 7-M and 4-W samples. Correlations > 0.70 are shaded. Correlations 0.5–0.7 are marked in bold-italic.

**Figure A5:**
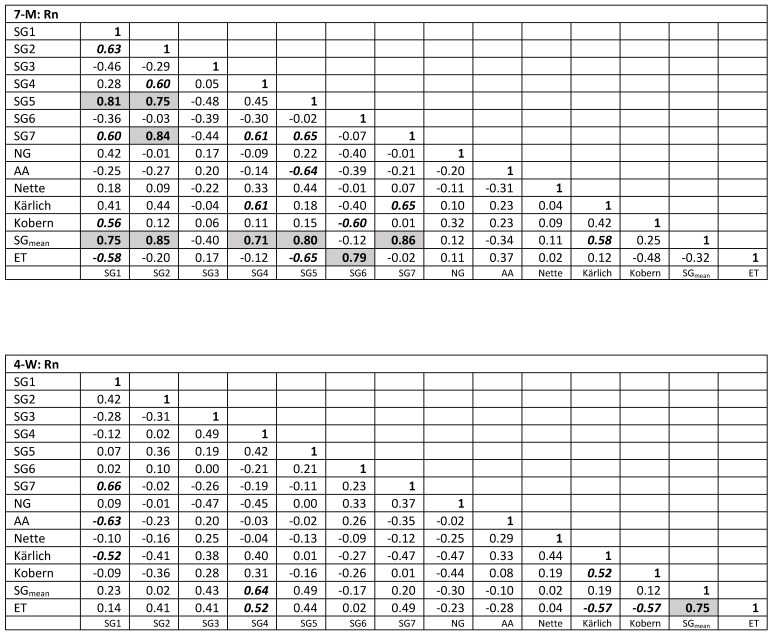
Correlation coefficients between Rn fluctuation patterns in NG, SG, SG_mean_, mineral springs, and Earth tides (ET) for the 7-M and 4-W samples. Correlations > 0.70 are shaded. Correlations 0.5–0.7 are marked in bold-italic.

Associations of semi-diurnal Earth tides with all gases from AA, NG and SG differed in the 7-M and 4-W samples (Appendix B, Figure A3, Figure A4 and Figure A5). Positively high (≈0.85; SG4, SG6) and moderate (0.57; SG_mean_) associations of Earth tides with CO_2_ fluctuation patterns were observed in the 7-M samples. A high negative influence of Earth tides on He was found for SG4 (−0.73; 7-M samples) and SG6 (−0.78; 4-W samples), whereas the relationship was strongly positive for Rn and Earth tides in SG6 (0.79; 7-M samples) and SG_mean_ (0.75; 4-W samples). Correlations between Earth tides and AA and NG were weak.

## 4. Discussion

### 4.1. Gases in Ambient Air, Nest and Soil

This is the first time that AA, NG and SG samples have been monitored in parallel in a long-term survey in the Neuwied Basin. The continual bi-weekly and 8-hour sampling intervals generated a robust geochemical data set for the Goloring site. Prior geochemical analyses in the EEVF were based only on annual [8] and short-term surveys of soil gases (4 days, [9]; Appendix G).

#### 4.1.1. CO_2_

Ohashi et al. [32] and Risch et al. [30] found that RWA nests are point sources of CO_2_ by measuring CO_2_ fluxes from RWA nests at 10-cm maximum depth. Ohashi et al. [32] suggested that surficial CO_2_ emissions from RWA nests originate from: (1) respiration processes of RWAs and other invertebrates within the nest; (2) root respiration by vascular plants within or beneath the nest; and (3) microbial decomposition of nest material. According to Hinkle [45], surficial gas samples from 0.0–0.8 m are influenced by the atmosphere. We took gas measurements at 1-m depth without atmospheric influence and the maximum NG concentrations (10.8 Vol. %; 2016) were comparable to others we took in June 2010 (≈15.0 Vol. %; Appendix G). CO_2_ degassing (measured at 1-m depth) from 2–100Vol. % can derive from deep fault zones and may be related to recent or post-volcanic metamorphic processes in carbonate rocks [46]. Our findings are comparable to ones of the Arabia Fault (8.2 Vol. %), and Terme S. Giovanni (18.1 Vol. %), a main thermal spring at the Rapolano Fault within the Neogene Siena-Radicofani Basin (Central Italy; [47]). We conclude that the NG results imply that the RWA nest is located above the fault core zone and indicate a degassing vent at the study site (Figure 1).

Most of the SG concentrations are slightly elevated, but the very high levels in SG5 and SG1 indicate CO_2_ anomalies (following [42]). Although higher ones have been reported (e.g., [9]), these are comparable to those recorded from the actively degassing Rapolano Fault [47] and therefore may be associated with an actively degassing but unknown fault on the Goloring site. Median concentrations were comparable to findings of actively degassing vents by Gal et al. [9] for the Laacher See pasture and Obermendig site, and to random samplings on the Goloring site (Appendix G).

#### 4.1.2. He

He concentrations lower than the atmospheric standard of 5.22 ppm ([18]; class I in Table 2) are considered to represent undisturbed background levels [48]; all other anomaly classes (II-V; Table 2) indicate tectonic influences.

He concentrations exceeding the atmospheric standard of 5.22 ppm ([18]; Table 2) were twice as high in NG than in SG in the 4-W samples, lending further support for the RWA nest being located above the core fault zone (Figure 1). The high NG concentrations support the notion that RWA nests are useful biological indicators for degassing faults at small, local scales [24,25,28]. This conclusion is further supported by the temporal analyses that indicate a coupled system of CO_2_-He in NG. Tectonically active zones are known for high He fluxes through permeable fractures. Compressive stress and seismic activity maintain permeabilities and lead to gas anomalies at the surface [10,16,49].

Differences between NG and SG1 concentrations may be attributed to different soil characteristics, different basement geology [9], variation in structure, damage intensity, and thickness along and between faults [36] or even to an unknown fault separating both locations. SG1, SG5, and SG7 probably are located on a less developed fault segment, for example, in the damaged zone, so that there is less permeability for fluid flow and degassing (Figure 1). Faults exhibiting minor gas emissions are often synthetic faults that root into main faults [50].

It was not possible to verify the validity of He concentrations below the atmospheric standard or its concentrations in AA. Such a validation of these low values would require a follow-up study to assess sampling or analytical errors.

#### 4.1.3. Rn

Although Rn concentrations at the Goloring site in AA and NG were within the background value (< 20 BqL^−1^; [43]), SG concentrations were up to 8-fold higher. Median SG concentrations differed up to 23-fold among sampling locations, confirming non-stationary variation in soil or bedrock at small scales [51]. In total, maximum Rn concentrations in SG at the Goloring site were up to 5-fold (7-M samples) or 2-fold higher (4-W samples) than those reported by Gal et al. [9] (Appendix G), indicating an increased Rn potential with a locally high potential (> 100 BqL^−1^) for the Goloring site [44]. Local high Rn anomalies (> 100 BqL^−1^) are associated with tectonic fault zones and clefts caused by advective gas transport along faults between the interbedding layers of Lower Devonian clay and siltstone bedrocks and the Cenozoic sediment basin fillings [52,53]. These concentrations also are comparable to hazardous sites along the Rapolano Fault [47]. The small-scale variability in Rn degassing at Goloring can be attributed to a linear fault-linked anomaly [10,13], suggesting a degassing in the NE-SW direction (Variscan direction) and the NW-SE direction (corresponding to the present-day main stress direction; [5]). Both accord with the local “Radon-Potential Map” [44]. Our data augment this “Radon-Potential Map”, complement knowledge of Rn anomalies in the Neuwied Basin, and extend information on geogenic radon potential for this area.

### 4.2. Time Series

Bi-weekly NG and SG samples were more variable than 4-W samples in the three environments. This supports our conclusion that results derived from samples taken at long intervals may lead to erroneous conclusions [28]. For example, Griesshaber [54] and Clauser et al. [7] concluded from annual samples that CO_2_ is the primary carrier in the Eifel fluid-rock system. Results of our higher-frequency samples cannot confirm this; we could only identify a CO_2_-He coupled system in NG and SG6 and a CO_2_-Rn coupled system in SG6 and SG7. Elsewhere, we hypothesized that geogenic gases in this part of the Neuwied Basin might be transported by another carrier gas, such as N_2_ ([28]; see also Bräuer et al. [8]). Bräuer et al. [8] found N_2_ to be a carrier gas at the periphery along the Rhine. Future investigations throughout the Neuwied Basin should investigate the N_2_-He carrier-trace gas couple also in soil gas samples.

### 4.3. External Factors

#### 4.3.1. Meteorological Conditions

The degassing processes from NG and SG did not appear to be influenced by meteorological conditions (cf. [9,45,55,56]). Additionally, our results do not support the hypothesis that temperature is a dominant controller of CO_2_ production (cf. [57]). The reason for these differences could be related to the frequency of measurements. We monitored them continuously on site where as others used daily values recorded at distant meteorological stations [9,57].

#### 4.3.2. Earthquakes

We observed no effects of earthquakes on gas concentrations in the biweekly (7-M) samples. This is attributable to either: (a) the small size of the earthquakes; (b) their large distance (>10 km) from the Goloring site; or that (c) biweekly sampling intervals missed the influence of the small earthquakes. Alternatively, evidence of seismic influence on fluctuation patterns were observed after the earthquake that occurred on 3 August 2016 at Nickenich (≈11 km) while we were sampling every 8 h. The decline of CO_2_, He, and Rn concentrations in NG and SG observed ≈1 day before the earthquake can be explained by: (1) an increase in compressive stress; (2) volume changes of the pore fluid or rock matrix; or (3) a permeability change of conduits at the nest and soil sample locations [16,49]. As there was only one such nearby event, however, we cannot assert that there is a general relationship between earthquakes and nest or soil gas concentrations. The recent occurrences of deep earthquakes that are related to magmatic processes at the lower crust suggest continuous monitoring in this youngest volcanic field in Germany. Such data also would help assess relationships between gas flux dynamics and earthquake events in the Neuwied Basin.

#### 4.3.3. Earth tides

Deformations of the Earth’s crust by Earth tides are associated with cyclic variations in water-table levels within the rock strata and have been suggested to influence gas concentrations (e.g., Rn; [58,23]). Though all NG and SG probes were ≤ 60 m from one another, we only observed an effect of Earth tides on the fluctuation patterns in 25% of the probes for the 7-M samples. This result could be explained by: (1) the 8-hour sampling interval being too long to capture effects of semi-diurnal earth tides; (2) the study area being too far away from coastlines, ameliorating influences of Earth tides; or (3) in the case of Rn, the emanating layer being located too deeply so that any pumped Rn is diluted during migration [23].

### 4.4. Comparison with Mineral Springs Nette, Kärlich and Kobern

A comparison of NG and SG concentrations with previously-investigated gas concentrations in nearby mineral springs [35] showed similar median He concentrations in SG, NG, and Kobern (Appendix D). We observed highly or very highly (CO_2_) to moderate (He) correlations in SG_mean_ and concentrations at Nette, Kärlich, and Kobern (Appendix B, Figure A3 and Figure A4). Similar median Rn concentrations were found for SG and Nette, Kärlich, and Kobern, suggesting degassing at Goloring site and the three springs are linked either by a similar Rn source in the subsurface or by an unknown fault system.

CO_2_ fluctuations in SG_mean_ and Nette and Kärlich mineral springs are directly and instantaneously linked. Correlations of He between SG_mean_ and Nette indicate a linkage in NW-SE direction and between SG_mean_ and Kärlich an additional linkage in NE-SW direction (Appendix E). This linkage between SG at Goloring site and the minerals springs is supported further by cross-correlations (Appendix F): SG_mean_ and Nette (lag ≈ 16 h) and Kärlich (lag ≈ 40 h) are either directly linked or positively correlated with respect to CO_2_. Moderate cross-correlation between SG_mean_ and Nette for He was observed (lag ≈ 3 days). Relations between NG and Nette and Kärlich (lag ≈ 80 h) were small and positive for CO_2_ and small and negative for He (lag ≈ 80 h to 5 days). NG and Kobern were related with a lag of ≈ 88 h for He. No to only low relations were observed for Rn between NG and the three springs (Appendix F).

Degassing rhythms in NG and SG were equivalently periodic and exhibited infradian rhythms of 2, 3, 4, and 6 days. The same infradian rhythms were found for the three mineral springs investigated [28]. These results support our conclusion that there are tectonic linkages between Goloring and Nette in the NW-SE direction (present-day stress field) and between Goloring and Kärlich in the NE-SW (Variscan fault direction) directions [28].

The volcanic activity in the EEVF is dormant but not extinct. Furthermore, information on active tectonic fault zones is missing in the EEVF and especially in the Ochtendunger Fault Zone. Monitoring of geogenic gases suggesting statistical bias when samples are taken at large temporal intervals. Therefore, we recommend daily soil gas samplings for a minimum of one year to understand—in combination with the recommended mineral water sampling—the EEVF’s tectonic, magmatic and degassing system also in relation to new developments in earthquake processes which are related to magmatic processes in the lower crust.

## 5. Conclusions

Combined analyses of ambient air (AA), ant-nest gases (NG), and soil gases (SG) measured in situ from 1 March–30 September 2016 were evaluated to determine composition, fluctuation patterns, temporal variations, degassing rhythms, and carrier-trace gas couples of geogenic gases (CO_2_, He, Rn,) and compared to gas concentrations in three nearby mineral springs. Results of continual sampling during 7 months (bi-weekly) and 4 weeks (every 8 h) were:

He concentrations in NG were above the atmospheric standard. A coupled CO_2_-He system supported the hypothesis that red wood-ant nests can be used as biological indicators for actively degassing faults.

Radon anomalies in SG with peak concentrations of 163 BqL^−1^ identified a high local Rn potential for the Goloring site and conributed to the Radon potential map of LGB-RLP 2017 [44].

Equivalently periodic degassing infradian rhythms in the red wood-ant nest, soil, and three nearby minerals springs suggested a NW-SE tectonic linkage between Goloring and Nette spring and a NE-SW tectonic linkage between Goloring and Kärlich spring.

Meteorology and low-magnitude local earthquakes did not modulate degassing at Goloring.

Analyses of fluctuation patterns revealed that only 25% of the probes were affected by Earth tides.

Earth tides were associated with soil degassing of CO_2_, He, and Rn only in biweekly samples, suggesting statistical bias when samples are taken at longer temporal intervals.

Because volcanic activity in the EEVF is dormant, more detailed information on active tectonic fault zones is needed in the EEVF, especially in the Ochtendunger Fault Zone. We recommend continuous monitoring of geogenic gases in soil and RWA nests—in combination with the recommended mineral water sampling and isotopic investigations—for a minimum of one year to understand the EEVF’s tectonic, magmatic, and degassing systems in relation to new developments in earthquake processes that are related to magmatic processes at the lower crust. 

Furthermore, electrical measurements, for example, between the tips of the probe in the nest on the fault and between other ones in the soil tens of meters away, could provide information about fluctuations of electronic charge carriers, which would be stress-activated at depths below, before, or during earthquakes.

## Figures and Tables

**Table 1:** Gas concentrations in ambient air (AA), NG, and SG for (a) 7-M and (b) 4-W samples.

		7-M (bi-weekly; 01.03.–30.09.2016) (a)							4-W (8-hrs. 12.07.–11.08.2016) (b)						
		N	Mean	Median	Min	Max	SD	Vol. %	N	Mean	Median	Min	Max	SD	Vol. %
**AA**	**CO_2_ (Vol. %)**	16	0.00	0.00	0.00	0.00	0.00	0.00	83	0.01	0.00	0.00	0.90	0.10	0.05
	**He (ppm)**	16	5.18	5.20	5.03	5.32	0.09	5.33	83	5.16	5.24	1.79	5.54	0.47	5.55
	**Rn (BqL^−1^)**	16	0.31	0.11	0.00	3.07	0.74	0.80	83	0.43	0.35	0.05	1.64	0.29	0.79
**NG**	**CO_2_ (Vol. %)**	16	0.20	0.00	0.00	0.80	0.30	0.50	83	0.64	0.40	0.00	10.80	1.56	1.58
	**He (ppm)**	16	5.11	5.17	4.09	5.27	0.28	5.45	83	5.17	5.22	2.90	5.85	0.32	5.49
	**Rn (BqL^−1^)**	16	1.77	1.63	0.05	6.06	1.53	3.90	83	5.85	5.09	0.19	15.65	4.39	12.57
**SG 1**	**CO_2_ (Vol. %)**	16	5.97	5.30	3.20	10.40	2.27	8.94	83	8.76	8.80	3.60	11.00	1.51	11.06
	**He (ppm)**	16	5.12	5.19	4.55	5.32	0.18	5.45	83	5.08	5.15	1.71	5.69	0.46	5.48
	**Rn (BqL^−1^)**	16	86.30	100.49	11.07	138.33	33.75	156.46	83	64.50	67.03	0.32	96.81	17.91	89.25
**SG 2**	**CO_2_ (Vol. %)**	16	3.58	3.20	0.60	6.60	1.72	5.79	83	4.06	4.20	0.80	5.80	0.88	5.29
	**He (ppm)**	16	5.23	5.21	5.11	5.45	0.09	5.37	83	5.21	5.21	5.07	5.35	0.06	5.30
	**Rn (BqL^−1^)**	16	19.78	20.74	5.89	29.68	6.83	31.81	83	12.77	12.56	0.10	59.79	6.59	18.97
**SG 3**	**CO_2_ (Vol. %)**	16	0.90	0.40	0.00	6.00	1.56	2.23	83	1.12	1.20	0.00	1.60	0.18	1.45
	**He (ppm)**	16	5.12	5.12	4.63	5.50	0.22	5.41	83	5.21	5.19	4.60	10.40	0.62	5.56
	**Rn (BqL^−1^)**	16	2.96	2.64	0.68	7.17	1.61	5.04	83	3.92	3.40	0.19	43.66	4.85	7.13
**SG 4**	**CO_2_ (Vol. %)**	16	2.50	2.40	0.60	4.20	1.13	4.33	83	3.70	3.80	1.40	4.40	0.48	4.42
	**He (ppm)**	16	5.16	5.18	4.75	5.35	0.13	5.34	83	5.29	5.18	1.77	11.18	0.99	5.82
	**Rn (BqL^−1^)**	16	113.83	118.00	53.75	162.90	33.28	172.76	83	92.38	98.58	23.81	145.71	26.99	141.84
**SG 5**	**CO_2_ (Vol. %)**	16	10.39	10.00	6.40	15.00	2.48	14.00	83	12.77	12.50	3.60	14.90	1.30	13.89
	**He (ppm)**	16	5.01	5.06	4.50	5.23	0.21	5.32	83	5.12	5.11	4.76	5.54	0.11	5.27
	**Rn (BqL^−1^)**	16	74.46	79.87	40.68	99.79	16.18	107.16	83	55.17	57.28	12.47	74.91	12.63	76.41
**SG 6**	**CO_2_ (Vol. %)**	16	0.75	0.60	0.00	1.80	0.51	1.39	83	1.05	1.00	0.00	1.40	0.24	1.36
	**He (ppm)**	16	5.16	5.15	5.00	5.25	0.07	5.25	83	5.25	5.17	4.88	11.10	0.67	5.50
	**Rn (BqL^−1^)**	16	8.65	7.87	2.78	14.31	3.09	12.67	83	11.69	10.63	0.72	64.55	8.43	17.48
**SG 7**	**CO_2_ (Vol. %)**	16	3.95	3.80	1.80	6.60	1.46	6.14	83	4.61	4.60	3.20	6.40	0.91	6.12
	**He (ppm)**	16	5.20	5.20	4.93	5.55	0.15	5.39	83	5.10	5.16	1.69	5.33	0.40	5.42
	**Rn (BqL^−1^)**	16	48.29	41.04	0.13	94.42	27.51	89.05	83	32.17	31.17	0.57	69.08	12.51	50.26

**Table 2:** Concentration classes of gases for AA, NG and SG for the 4-W sampling. CO_2_ classes after [42]; atmospheric He after [18]; and Rn background concentrations after [43] and [44].

Classes	AA	NG	SG1	SG2	SG3	SG4	SG5	SG6	SG7
**(a) CO_2_ (Vol. %) ^1^**									
I: <1	100.0	96.4	0.0	0.0	2.4	0.0	0.0	1.2	0.0
II: 1–9.99	0.0	2.4	74.7	100	97.6	100	1.2	97.6	100
III: >10	0.0	1.2	25.3	0.0	0.0	0.0	98.8	1.2	0.0
Sum (%)	100.0	100.0	100.0	100.0	100.0	100.0	100.0	100.0	100.0
**(b) He (ppm)**									
I: <5.22 ^2^ (undisturbed background levels)	39.8	50.6	84.3	60.2	59.0	77.1	85.5	80.7	86.7
II: 5.22–5.29	48.2	38.6	12.0	33.8	30.1	14.5	10.8	15.7	12.0
III: 5.30–5.39	9.6	7.2	1.2	6.0	9.6	2.4	1.2	1.2	1.3
IV: 5.40–5.59	2.4	2.4	1.2	0.0	0.0	2.4	2.5	0.0	0.0
V: >5.60	0.0	1.2	1.3	0.0	1.3	3.6	0.0	2.4	0.0
Sum (%)	100.0	100.0	100.0	100.0	100.0	100.0	100.0	100.0	100.0
Sum (%) >5.22 ppm	60.2	49.4	15.7	39.8	41.0	22.9	14.5	19.3	13.3
**(c) Rn (BqL^−1^)**									
I: <20 (background concentration) ^3^	100	100	3.6	98.8	98.8	0.0	1.2	96.3	15.7
II: 20–39.9 (low-moderate) ^3^	0.0	0.0	3.6	0.0	0.0	4.8	9.6	0.0	57.8
III: 40–99.9 (increased with locally high potential > 100 BqL^−1^) ^3^	0.0	0.0	92.8	1.2	1.2	49.3	89.2	3.7	26.5
IV: >100 (locally high potential) ^4^	0.0	0.0	0.0	0.0	0.0	45.9	0.0	0.0	0.0
Sum (%)	100.0	100.0	100.0	100.0	100.0	100.0	100.0	100.0	100.0

^1^ Concentration classes according to [42]; ^2^ Atmospheric standard: 5.2204 ±0.0041ppm [18]; ^3^ Background concentration [43]; ^4^ Rn potential classes [44].

**Figure A6:**
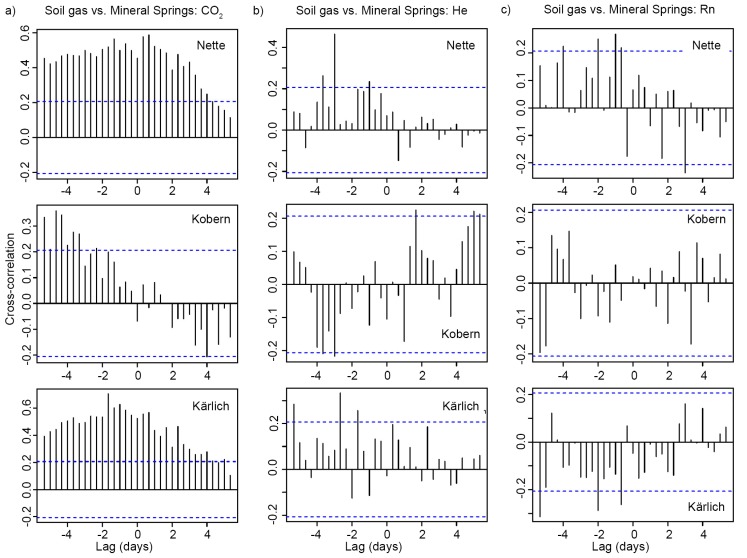
Cross-correlation of the time-series of SG_mean_ vs. mineral springs (8-hour median smoothed) for (**a**) CO_2,_ (**b**) He, and (**c**) Rn for the 4-W samples. Blue dashed lines indicate confidence thresholds.

**Figure A7:**
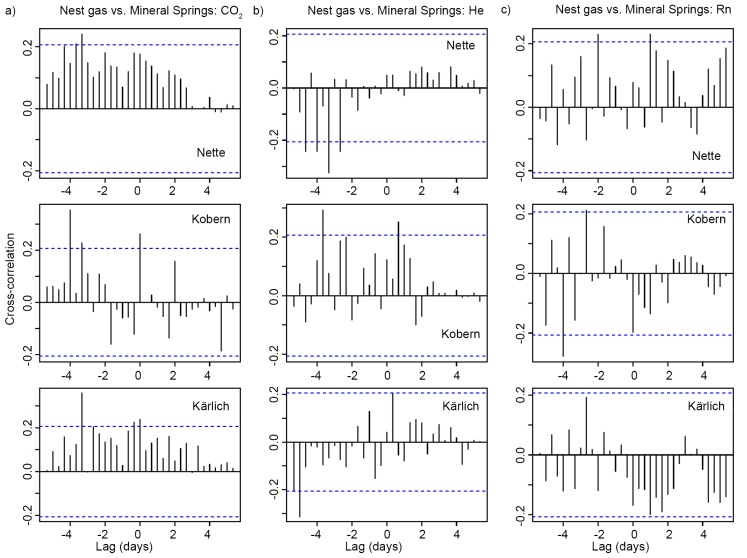
Cross-correlation of the time-series of NG vs. mineral springs (8-hour median smoothed) for (**a**) CO_2,_ (**b**) He, and (**c**) Rn for the 4-W samples. Blue dashed lines indicate confidence thresholds.

## Appendix C

**Table A1:** Modified Fourier analysis for CO_2_ in AA, NG and SG 1-7 (4-W samples). Maxima of amplitude are highlighted in bold.

Days	CO_2_ AA	CO_2_ NG	CO_2_ SG1	CO_2_ SG2	CO_2_ SG3	CO_2_ SG4	CO_2_ SG5	CO_2_ SG6	CO_2_ SG7
**1**									
**2**	0.2	5.0	5.1	2.7	0.5	1.3	1.5	4.2	1.4
**3**	0.2	**6.2**	7.2	3.5	0.4	1.6	1.4	4.1	2.3
**4**	0.2	5.1	9.6	2.5	0.6	1.6	1.5	4.1	
**5**		4.8		4.5				3.6	4.8
**6**	0.2	3.9	9.4	4.2	0.7	0.7	1.0	4.1	4.2
**7**			11.1		0.5	1.4		4.0	
**8**	0.2						1.3		4.3
**9**				4.7		**2.3**			
**10**		3.1						**4.4**	
**11**			11.7						
**12**									
**13**	0.2			**5.4**	0.8		0.5		3.7
**14**									
**15**								4.2	
**16**									
**17**									
**18**		4.2			**1.0**		1.3		
**19**									
**20**									
**21**									
**22**			**13.8**			1.2		4.2	
**23**									
**24**									
**25**									
**26**									
**27**									
**28**									
**29**									
**30**							**2.1**		**5.0**

**Table A2:** Modified Fourier analysis for He in AA, NG and SG 1-7 (4-W samples). Maxima of amplitude are highlighted in bold.

Days	He AA	He NG	He SG1	He SG2	He SG3	He SG4	He SG5	He SG6	He SG7
**1**	686.7	433.0	403.9	280.6	701.4	1059.3	307.5	3381.8	565.8
**2**	676.7	767.0	756.2	405.9	910.4	**1370.2**	538.6	4115.7	806.3
**3**	**984.0**	529.1	691.0	374.0	676.0	1338.3	638.4	5832.6	900.5
**4**	833.9	694.7	818.6	318.3	828.3	1073.4	622.2	6504.7	574.6
**5**	784.6	789.5	1.142.4	307.1	837.6	1252.1	817.8		622.1
**6**	802.0	**879.3**	1.162.9	**491.5**			725.8		843.0
**7**								6.630.6	
**8**						1335.4			1.101.0
**9**		823.1		108.5	551.5			6.705.9	
**10**	802.5		983.3						872.1
**11**									
**12**		622.3		175.0		862.1	794.3	6.771.3	
**13**									559.6
**14**									
**15**									
**16**									
**17**	624.8	702.5							
**18**									
**19**									
**20**									
**21**				436.3		840.2	**1267.7**	**7296.0**	**1273.0**
**22**									
**23**									
**24**									
**25**									
**26**									
**27**									
**28**									
**29**			**1.421.4**						
**30**	686.7	433.0	403.9	280.6	701.4	1.059.3	307.5	3.381.8	565.8
**…**									
**45**				362.3	**2.087.6**		1.046.9		

**Table A3:** Modified Fourier analysis for Rn in AA, NG and SG 1-7 (4-W samples). Maxima of amplitude are highlighted in bold.

Days	Rn AA	Rn NG	Rn SG1	Rn SG2	Rn SG3	Rn SG4	Rn SG5	Rn SG6	Rn SG7
**1**	1.5	17.2	51.1	34.9	12.0	62.0	53.9	40.1	28.3
**2**	1.5	**47.2**		55.3	16.7	188.3	35.6	59.8	64.5
**3**	2.6	37.4	164.9	47.3	10.7	211.8	95.7	49.1	75.3
**4**	1.5	21.9	143.2	75.6	13.4	67.7	109.3		67.3
**5**	1.8	21.0		54.5	**19.5**		40.9	48.3	60.9
**6**	2.1	9.9	101.0						
**7**		17.8			11.8				
**8**				45.4		128.6		58.1	
**9**		47.3							55.6
**10**	2.5				12.4			**62.5**	
**11**									
**12**				**77.3**					
**13**			**218.3**			141.4	129.6		
**14**	**2.6**								**92.9**
**15**									
**16**									
**17**		37.8		70.6				40.7	
**18**									
**19**									
**20**									
**21**					11.2				
**22**									
**23**									
**24**									
**25**									
**26**									
**27**									
**28**									
**29**	0.9								
**30**	1.5	17.2	51.1	34.9	12.0	62.0	53.9	40.1	28.3
**…**									
**41**							**129.6**		
**…**									
**45**				58.7		**426.5**			

## Appendix D

**Table A4:** Comparison of median He and Rn concentrations in NG, SG, and mineral springs for (a) the 7-M and (b) 4-W samples. Data for mineral springs are extracted from Berberich et al. (2017 [35]). Data for NG and SG from this study.

		7-M (bi-weekly; 01.03.–30.09.2016) (a)							4-W (8-hrs. 12.07.–11.08.2016) (b)						
		N	Mean	Median	Min	Max	SD	Vol. %	N	Mean	Median	Min	Max	SD	Vol. %
**Nette**	**He (ppm)**	16	47.06	49.74	17.37	58.17	10.91	63.63	79	49.20	50.29	20.83	110.40	13.24	67.55
	**Rn (Bq/L)**	16	6.47	6.26	2.62	9.46	1.88	9.11	79	8.01	6.58	0.56	59.90	8.08	12.92
**Kärlich**	**He (ppm)**	16	7.77	7.80	6.21	9.03	839.47	9.16	79	7.84	7.77	6.02	10.95	0.82	9.06
	**Rn (Bq/L)**	16	72.44	73.69	38.32	114.02	15.22	92.00	79	73.73	78.11	4.91	92.33	15.58	99.71
**Kobern**	**He (ppm)**	16	2.97	2.02	0.98	1.11	2.55	5.56	79	4.38	4.48	1.00	7.50	1.24	6.23
	**Rn (Bq/L)**	16	45.78	46.63	16.90	64.84	11.41	62.48	79	48.66	49.97	7.82	79.78	9.67	61.47
**NG**	**He (ppm)**	16	5.11	5.17	4.09	5.27	0.28	5.45	83	5.17	5.22	2.90	5.85	0.32	5.49
	**Rn (Bq/L)**	16	1.77	1.63	0.05	6.06	1.53	3.90	83	5.85	5.09	0.19	15.65	4.39	12.57
**SG1**	**He (ppm)**	16	5.12	5.19	4.55	5.32	0.18	5.45	83	5.08	5.15	1.71	5.69	0.46	5.48
	**Rn (Bq/L)**	16	86.30	100.49	11.07	138.33	33.75	156.46	83	64.50	67.03	0.32	96.81	17.91	89.25
**SG2**	**He (ppm)**	16	5.23	5.21	5.11	5.45	0.09	5.37	83	5.21	5.21	5.07	5.35	0.06	5.30
	**Rn (Bq/L)**	16	19.78	20.74	5.89	29.68	6.83	31.81	83	12.77	12.56	0.10	59.79	6.59	18.97
**SG3**	**He (ppm)**	16	5.12	5.12	4.63	5.50	0.22	5.41	83	5.21	5.19	4.60	10.40	0.62	5.56
	**Rn (Bq/L)**	16	2.96	2.64	0.68	7.17	1.61	5.04	83	3.92	3.40	0.19	43.66	4.85	7.13
**SG4**	**He (ppm)**	16	5.16	5.18	4.75	5.35	0.13	5.34	83	5.29	5.18	1.77	11.18	0.99	5.82
	**Rn (Bq/L)**	16	113.83	118.00	53.75	162.90	33.28	172.76	83	92.38	98.58	23.81	145.71	26.99	141.84
**SG5**	**He (ppm)**	16	5.01	5.06	4.50	5.23	0.21	5.32	83	5.12	5.11	4.76	5.54	0.11	5.27
	**Rn (Bq/L)**	16	74.46	79.87	40.68	99.79	16.18	107.16	83	55.17	57.28	12.47	74.91	12.63	76.41
**SG6**	**He (ppm)**	16	5.16	5.15	5.00	5.25	0.07	5.25	83	5.25	5.17	4.88	11.10	0.67	5.50
	**Rn (Bq/L)**	16	8.65	7.87	2.78	14.31	3.09	12.67	83	11.69	10.63	0.72	64.55	8.43	17.48
**SG7**	**He (ppm)**	16	5.20	5.20	4.93	5.55	0.15	5.39	83	5.10	5.16	1.69	5.33	0.40	5.42
	**Rn (Bq/L)**	16	48.29	41.04	0.13	94.42	27.51	89.05	83	32.17	31.17	0.57	69.08	12.51	50.26

## Appendix G

**Table A5:** Comparison of gas concentrations in SG gas (a) from gas vents at Laacher See pasture (2007; [9]); (b) Obermendig Site (2007; [9]); (c) random samplings at Goloring site (2010; Berberich. unpublished); and average values of SG1–SG7 (SG_mean_) of the 7-M (d) and 4-W samples (e) at Goloring (2016; this study). n.d. = not determined.

		N	Mean	Median	Min	Max	SD	Vol. %
**(a) Laacher See pasture (24.9–27.9.2007; [9])**								
**SG**	**CO_2_ (Vol. %)**	87	13.80	3.30	0.03	100.00	23.70	n.d.
	**He (ppm)**	87	5.44	5.25	1.33	10.10	1.00	n.d.
	**Rn (BqL^−1^)**	70	8.63	6.96	0.12	30.80	6.84	n.d.
**(b) Obermendig Site (24.9–27.9. 2007; [9])**								
**SG**	**CO_2_ (Vol. %)**	12	14.50	5.30	0.13	90.00	24.00	n.d.
	**He (ppm)**	12	4.93	5.16	2.75	5.26	0.67	n.d.
	**Rn (BqL^−1^)**	12	24.50	18.30	0.33	81.00	24.50	n.d.
**(c) Random samplings at Goloring site (29.4.2010; Berberich. unpublished)**								
**SG**	**CO_2_ (Vol. %)**	9	4.18	2.00	1.20	13.00	4.39	8.69
	**He (ppm)**	9	5.16	5.18	5.07	5.25	67.50	5.23
	**Rn (BqL^−1^)**	9	32.02	19.24	0.06	91.18	29.27	65.58
**(d) Goloring site 7-M (bi-weekly; 01.03.–30.09.2016; this study)**								
**SG_mean_**	**CO_2_ (Vol. %)**	112	4.03	3.20	0.00	15.00	3.55	8.74
	**He (ppm)**	112	5.14	5.17	4.50	5.55	1.67	5.38
	**Rn (BqL^−1^)**	112	50.61	34.46	0.13	162.90	44.83	112.85
**(e) Goloring site 4-W (8-hrs; 12.07.–11.08.2016; this study)**								
**SG_mean_**	**CO_2_ (Vol. %)**	630	5.17	4.00	0.00	14.90	4.03	10.60
	**He (ppm)**	83	5.18	5.17	1.69	11.18	5.52	5.45
	**Rn (BqL^−1^)**	83	39.03	29.36	0.10	145.71	33.64	87.44

## References

[1] Wörner G. (1998). Quaternary Eifel volcanism, its mantle sources and effect on the crust of the Rhenish Shield. Young Tectonics–Magmatism–Fluids, a Case Study of the Rhenish Massif.

[2] Ritter J.R.R., Jordan M., Christensen U., Achauer U. (2001). A mantle plume below the Eifel volcanic fields, Germany. Earth Planet. Sci. Lett..

[3] Schmincke H.U. (2007). The Quaternary volcanic fields of the East and the West Eifel (Germany). Mantle Plumes.

[4] Ahorner L., Fuchs K., von Gehlen K., Mälzer H., Murawski H., Semmel A. (1983). Historical seismicity and present-day microearthquake activity in the Rhenish Massif, Central Europe. Plateau Uplift: The Rhenish Shield—A Case History.

[5] Hinzen K.G. (2003). Stress field in the Northern Rhine area, Central Europe, from earthquake fault plane solutions. Tectonophysics.

[6] May F. (2002). Quantifizierung des CO_2_-Flusses zur Abbildung magmatischer Prozesse im Untergrund der Westeifel.

[7] Clauser C., Griesshaber E., Neugebauer H.J. (2002). Decoupled thermal and mantle helium anomalies: Implications for the transport regime in continental rift zones. J. Geophys. Res..

[8] Bräuer K., Kämpf H., Niedermann S., Strauch G. (2013). Indications for the existence of different magmatic reservoirs beneath the Eifel area (Germany): A multi-isotope (C, N, He, Ne, Ar) approach. Chem. Geol..

[9] Gal F., Brach M., Braibant G., Jouin F., Michel K. (2011). CO_2_ escapes in the Laacher See region, East Eifel, Germany: application of natural analogue onshore and offshore geochemical monitoring. Int. J. Greenhouse Gas. Control..

[10] Baubron J.C., Rigo A., Toutain J.P. (2002). Soil gas profiles as a tool to characterize active tectonic areas: The Jaut Pass example (Pyrenees, France). Earth Planet. Sci. Lett..

[11] Wilkinson M., Gilfillan S.M.V., Haszeldine R.S., Ballentine C.J. (2009). Plumbing the depths: Testing natural tracers of subsurface CO_2_ origin and migration, Utah. Carbon Dioxide Sequestration in Geological Media–State of the Science.

[12] Ciotoli G., Etiope G., Guerra M., Lombardi S. (1999). The detection of concealed faults in the Ofanto basin using the correlation between soil-gas fracture surveys. Tectonophysics.

[13] Ciotoli G., Lombardi S., Zarlenga F. (2006). Natural leakage of helium from Italian sedimentary basins of the Adriatic structural margin. Perspectives for geological sequestration of carbon dioxide. Advances in the Geological Storage of Carbon Dioxide.

[14] Etiope G. (2009). Natural emissions of methane from geological seepage in Europe. Atmos. Environ..

[15] Voltattorni N., Sciarra A., Quattrocchi F. The Application of Soil-Gas Technique to Geothermal Exploration: Study of Hidden Potential Geothermal Systems. Proceedings of the World Geothermal Congress.

[16] Birdsell D.T., Rajaram H., Dempsey D., Viswanathan H.S. (2015). Hydraulic fracturing fluid migration in the subsurface: A review and expanded modeling results. Water Resour. Res..

[17] Giroud N., Vuataz F.D., Schill E. Permeable Fault Detection in Deep Geothermal Aquifer Exploration by Soil Gas Measurement. Proceedings of the Symposium 2: Structural Geology, Tectonics and Geodynamics.

[18] Davidson T.A., Emerson D.E. (1990). Direct determination of the helium 3 content of atmospheric air by mass spectrometry. J. Geophys Res. Atmosph..

[19] Kockarts G. (1973). Helium in the terrestrial atmosphere. Space Sci. Rev..

[20] Sano Y., Furukawa Y., Takahata N. (2010). Atmospheric helium isotope ratio: Possible temporal and spatial variations. Geochim. Cosmochim. Acta.

[21] Gilfillan S.M.V., Wilkinson M., Haszeldinea R.S., Shiptonb Z.K., Nelsonc S.T., Poredad R.J. (2011). He and Ne as tracers of natural CO_2_ migration up a fault from a deep reservoir. Int. J. Greenhouse Gas. Control..

[22] Ciotoli G., Lombardi S., Morandi S., Zarlenga F. (2004). A multidisciplinary statistical approach to study the relationships between helium leakage and neo-tectonic activity in a gas province: The Vasto Basin, Abruzzo-Molise (Central Italy). AAPG Bull.

[23] Crockett R.G.M., Gillmore G.K., Phillips P.S., Denman A.R., Groves-Kirkby C.J. (2006). Tidal synchronicity of built-environment radon levels in the UK. Geophys. Res. Let..

[24] Berberich G. (2010). Identifikation Junger Gasführender Störungszonen in der West–und Hocheifel Mit Hilfe von Bioindikatoren. Ph.D. Thesis.

[25] Berberich G., Schreiber U. (2013). GeoBioScience: Red Wood Ants as Bioindicators for Active Tectonic Fault Systems in the West Eifel (Germany). Animals.

[26] Berberich G., Berberich M., Grumpe A., Wöhler C., Schreiber U. (2013). First Results of 2.5 Year Monitoring of Red Wood Ants’ Behavioural Changes and Their Possible Correlation with Earthquake Events. Animals.

[27] Berberich G., Grumpe A., Berberich M., Klimetzek D., Wöhler C. (2016). Are red wood ants (Formica rufa-group) tectonic indicators? A statistical approach. Ecol. Ind..

[28] Berberich G.M., Ellison A.M., Berberich M.B., Grumpe A., Becker A., Wöhler C. (2018). Can a Red Wood-Ant Nest Be Associated with Fault-Related CH4 Micro-Seepage? A Case Study from Continuous Short-Term In-Situ Sampling. Animals.

[29] Del Toro I., Berberich G.M., Ribbons R.R., Berberich M.B., Sanders N.J., Ellison A.M. (2017). Nests of red wood ants (*Formica rufa*-group) are positively associated with tectonic faults: A double-blind test. PeerJ.

[30] Risch A.C., Jurgensen M.F., Schütz M., Page-Dumroese D.S. (2005). The contribution of red wood ants to soil C and N pools and CO_2_ emissions in subalpine forests. Ecology.

[31] Risch A.C., Schütz M., Jurgensen M.F., Domisch T., Ohashi M., Finér L. (2005). CO_2_ emissions from red wood ant (*Formica rufa* group) mounds: Seasonal and diurnal patterns related to air temperature. Ann. Zool. Fennici.

[32] Ohashi M., Finér L., Domisch T., Risch A.C., Jurgensen M.F. (2005). CO_2_ efflux from a red wood ant mound in a boreal forest. Agric. For. Meteorol..

[33] Ohashi M., Finér L., Domisch T., Risch A.C., Jurgensen M.F., Niemelä P. (2007). Seasonal and diurnal CO_2_ efflux from red wood ant (*Formica aquilonia*) mounds in boreal coniferous forests. Soil Biol. Biochem..

[34] Wu H., Lu X., Wu D., Song L., Yan X., Liu J. (2013). Ant mounds alter spatial and temporal patterns of CO_2_, CH_4_ and N_2_O emissions from a marsh soil. Soil Biol. Biochem..

[35] Berberich G.M., Berberich M.B., Ellison A.M. (2017). Fluctuations of gas concentrations in three mineral springs of the East Eifel Volcanic field (EEVF). arXiv.

[36] Chester F.M., Evans J.P., Biegel R.L. (1993). Internal Structure and Weakening Mechanisms of the San Andreas Fault. J. Geophys. Res..

[37] BNS–Erdbebenstation Bensberg. www.seismo.uni-koeln.de/catalog/index.htm.

[38] Reimann C., Filzmoser P., Garrett R.G. (2005). Background and threshold: Critical comparison of methods of determination. Sci. Total Environ..

[39] Hinkle D.E., Wiersma W., Jurs S.G. (2009). Applied Statistics for the Behavioral Sciences.

[40] Milbert D. (2016). Solid Earth Tide. http://geodesyworld.github.io/SOFTS/solid.htm.

[41] Oppenheim A.V., Schafer R.W., Buck J.R. (1999). Discrete-Time Signal Processing.

[42] Sauer U., Watanabe N., Singh A., Dietrich P., Kolditz O., Schütze C. (2014). Joint interpretation of geoelectrical and soil-gas measurements for monitoring CO_2_ releases at a natural analogue. Near Surface Geophys..

[43] Kemski J., Siehl A., Stegmann R., Valdivia-Manchego M. Geogene Faktoren der Strahlenexposition unter besonderer Berücksichtigung des Radonpotentials. Schriftenreihe Reaktorsicherheit und Naturschutz.

[44] LGB-RLP (2017) Radonpotentialkarte der Osteifel Landesamt für Geologie und Bergbau Rheinland-Pfalz, Ausdruck vom. http://www.lgb-rlp.de/karten-und-produkte/online-karten/online-karte-radonprognose.html..

[45] Hinkle M.E. (1994). Environmental conditions affecting concentrations of He, CO_2_, O_2_, and N_2_ in soil gases. Appl. Geochem..

[46] Pfanz H. (2008). Mofetten. Kalter Atem schlafender Vulkane.

[47] Etiope G., Guerra M., Raschi A. (2005). Carbon Dioxide and Radon Geohazards Over a Gas-bearing Fault in the Siena Graben (Central Italy). TAO.

[48] Risdianto D., Kusnadi D. The Application of a Probability Graph in Geothermal Exploration. Proceedings of the World Geothermal Congress 2010.

[49] Boothroyd I.M., Almond S., Worrall F., Davies R.J. (2017). Assessing the fugitive emission of CH_4_ via migration along fault zones—Comparing potential shale gas basins to non-shale basins in the UK. STOTEN.

[50] Jolie E., Klinkmueller M., Moeck I. Diffuse Degassing Measurements in Geothermal Exploration of Fault Controlled Systems. Proceedings of the World Geothermal Congress 2015.

[51] Richon P., Perrier F., Jean E.-P., Sabroux C. (2009). Detectability and significance of 12 hr barometric tide in radon-222 signal, dripwater flow rate, air temperature and carbon dioxide concentration in an underground tunnel. Geophys. J. Int..

[52] Kemski J., Klingel R., Siehl A., Neznal M., Matolin N. Erarbeitung fachlicher Grundlagen zur Beurteilung der Vergleichbarkeit unterschiedlicher Messmethoden zur Bestimmung der Radonbodenluftkonzentration - Vorhaben 3609S10003. Bd. 2 Sachstandsbericht “Radonmessungen in der Bodenluft -Einflussfaktoren, Messverfahren, Bewertung”, 2012. https://doris.bfs.de/jspui/bitstream/urn:nbn:de:0221-201203237830/3/BfS_2012_3609S10003_Bd2.pdf.

[53] LGB RLP (2005). Geologie von Rheinland-Pfalz.

[54] Griesshaber E. (1998). The distribution pattern of mantle derived volatiles in mineral waters of the Rhenish Massif. Young Tectonics–Magmatism–Fluids, a Case Study of the Rhenish Massif.

[55] Toutain J.P., Baubron J.C. (1999). Gas geochemistry and seismotectonics: A review. Tectonophysics.

[56] Padilla G.D., Hernández P.A., Padrón E., Barrancos J., Pérez N.M., Melián G., Nolasco D., Dionis S., Rodríguez F., Calvo D. (2013). Soil gas radon emissions and volcanic activity at El Hierro (Canary Islands): The 2011-2012 submarine eruption, Geochem. Geophys. Geosyst..

[57] Beaubien S., Jones D., Gal F., Barkwith A., Braibant G., Baubron J.C., Ciotoli G., Graziani S., Lister T., Lombardi S. (2013). Monitoring of near-surface gasgeochemistry at the Weyburn, Canada, CO_2_-EOR site, 2001–2011. Int. J. Greenh. Gas. Control..

[58] Barnet I., Procházka J., Skalský L. (1997). Do the Earth tides have an influence on short-term variations in radon concentration?. Rad. Prot. Dos..

